# Core outcomes in periodontal trials: study protocol for core outcome set development

**DOI:** 10.1186/s13063-017-2169-z

**Published:** 2017-09-20

**Authors:** Thomas J. Lamont, Jan E. Clarkson, David N. J. Ricketts, Peter A. Heasman, Craig R. Ramsay

**Affiliations:** 1 Dundee Dental School, Park Place, University of Dundee, Dundee, UK; 2 School of Dental Sciences, Newcastle University, Newcastle upon Tyne, UK; 3 Health Services Research Unit, University of Aberdeen, Aberdeen, UK

**Keywords:** Periodontics, Delphi technique, Dentistry, Outcomes, Review, Clinical trial

## Abstract

**Background:**

There are a large number of clinical outcome measures used to assess the effectiveness of prevention and management strategies of periodontal diseases. This heterogeneity causes difficulties when trying to synthesise data for systematic reviews or clinical guidelines, reducing their impact. Core outcome sets are an agreed, standardised list of outcomes that should be measured and reported in all trials in specific clinical areas. We aim to develop a core outcome set for effectiveness trials investigating the prevention and management of periodontal disease in primary or secondary care.

**Methods:**

To identify existing outcomes we screened the Cochrane systematic reviews and their included studies on the prevention and management of periodontal diseases. The core outcome set will be defined by consensus of key stakeholders using an online e-Delphi process and face-to-face meeting. Key stakeholders involved in the development will include: patients, dentists, hygienists/therapists, specialists, clinical researchers and policy-makers. Stakeholders will be asked to prioritise outcomes and feedback will be provided in the next round(s). Stakeholders will have an opportunity to add outcomes found in the Cochrane review screening process at the end of the first round. If consensus is not reached after the second round we will provide feedback prior to a third round. Remaining outcomes will be discussed at a face-to-face meeting and agreement will be measured via defined consensus rules of outcome inclusion.

**Discussion:**

The inclusive consensus process should provide a core outcome set that is relevant to all key stakeholders. We will actively disseminate our findings to help improve clinical trials, systematic reviews and clinical guidelines with the ultimate aim of improving the prevention and management of periodontal diseases.

**Trial registration:**

COMET (http://www.comet-initiative.org/studies/details/265?result=true). Registered on August 2012.

## Background

Periodontal (gum) disease and caries (tooth decay) are the world’s most prevalent noncommunicable diseases. These diseases are largely preventable, yet they remain the major cause of poor oral health worldwide affecting an estimated four billion individuals in 2010 [1–3].

Periodontal disease affects tissues surrounding and supporting the teeth and is classified into two broad categories: gingivitis and periodontitis. Gingivitis is a reversible condition characterised by inflammation and bleeding of the gingiva. Periodontitis is the irreversible destruction and loss of the supporting periodontal structures (periodontal ligament, cementum and alveolar bone). The result is often gingival recession, sensitivity of the exposed root surface, root caries (decay), mobility and drifting of teeth and, ultimately, tooth loss. Periodontal disease is the primary cause of tooth loss in older adults [4, 5]. Untreated caries in permanent teeth is ranked as the most prevalent global condition with severe periodontal disease ranked sixth [6].

Periodontal disease shares common risk factors with other chronic diseases and conditions, such as obesity, heart disease, stroke, cancer, chronic obstructive pulmonary disease and diabetes. These include age, diet, physical inactivity, stress, immune system susceptibility, smoking and alcohol use. It is also recognised that there can be a bidirectional relationship between periodontal disease and other diseases. For example, recent research shows that chronic periodontitis has an adverse effect on the control of blood sugar, and the incidence of diabetic complications. Periodontal disease has also been associated with rheumatoid arthritis, coronary heart disease and adverse pregnancy outcomes [7–10]. Whilst causation has not been proven, the prevention and management of periodontal disease for these individuals is considered important for general wellbeing and quality of life.

Recent clinical guidance and systematic reviews have highlighted the lack of high-quality evidence regarding the prevention and management of periodontal disease [11–13]. Part of the reason for this is the large number of outcomes and clinical indices that are measured within the trials comparing the effectiveness of the management options. This heterogeneity of outcome measures has created difficulties when trying to compare or combine different study results leading to the confusion of patients and clinicians when considering the best treatment options. There is currently no consensus as to which outcomes should be measured when investigating interventions for periodontal disease.

The COMET (Core Outcome Measures in Effectiveness Trials) initiative (http://www.comet-initiative.org) brings together researchers interested in the development, application and promotion of core outcome sets (COS), that are defined as an agreed, standardised collection of outcomes which should be measured and reported in all trials for a specific clinical area. They represent the minimum that should be measured and reported upon in all trials, and are also suitable for other types of research and audit.

The COMET initiative maintains a database of completed and ongoing COS protocols and documents that are freely available to the public. This project has been registered on the COMET database.

Developing a COS for periodontal disease will encourage clinical researchers/trialists to use outcomes chosen by consensus that are relevant to patients and clinicians and help data synthesis and dissemination in the future.

### Objectives and scope

We will lead the development of a COS for the management of periodontal disease. The outcome set will be applicable to effectiveness trials investigating the prevention and management of periodontal disease in primary or secondary care. It will not be limited by health status, age or the geographical location of trials.

The core set will be developed for the prevention and management of periodontal diseases due to the overlap of their traditional management strategies, e.g. oral-hygiene aids and interventions are important aspects of both.

## Methods

### Identifying existing knowledge

To find potentially relevant outcome domains in the current academic literature we searched the Cochrane database of systematic reviews for relevant published reviews and protocols investigating the prevention and treatment of periodontal disease. The search was conducted up to July 2016. From the reviews and protocols that met our inclusion criteria we recorded the type of intervention(s), outcome measures (clinical, patient and economic) and duration of follow-up that the authors stated that they would investigate. We subsequently extracted data from all the trials within the included reviews and recorded additional outcome measures and indices reported by these trials. One investigator (TL) independently extracted the data and the results were reviewed by a second investigator (JC). Results were tabulated using Microsoft Office. A list of unique outcome measures reported was compiled.

### Consensus process

The COS will be developed by a consensus process involving key stakeholders, namely patients and dental professionals as well as clinical researchers, health economists, statisticians, policy-makers and industry representatives with experience in dental research. Patient participants will be recruited from across Scotland using the SHARE network and dental professionals and researchers will be contacted via professional bodies. To increase COS uptake, we will engage with the Cochrane Oral Health Group, clinical guideline developers, research funders, journal editors, regulators, such as research ethics committees, and trial registries. We will attempt to ensure adequate representation from each of the stakeholder groups to improve the acceptance and implementation of the COS. We will attempt to gain consensus via an online e-Delphi process which was chosen as the most efficient and pragmatic method of including the key stakeholders from different settings, backgrounds and geographical locations.

Stakeholders will be sent an invitation (plain language version sent where appropriate) with the background of the COS development process provided with additional information regarding the practicalities of the consensus process. Participants will be asked to opt in by replying to the invitation. To minimise attrition, only those stakeholders confirming that they would like to be involved in the process will be invited to complete the first round.

Outcomes will be presented in lay terms with the dental terms in brackets to ensure that all participants understand the terminology. The outcomes will be presented in alphabetical order and the participants will be asked to score each outcome from the combined long list using the scale proposed by the GRADE group (http://www.gradeworkinggroup.org), in which 1 to 3 signifies an outcome of limited importance, 4 to 6 important but not critical, and 7 to 9 critical.

Reminders will be sent before the end of each round to those participants who completed the previous round but have not yet responded by 2 weeks after invitation. At the end of the first round participants will be given the opportunity to suggest any outcome measures that they would consider relevant but missing from the list of outcomes. They will be informed that a minimum of two participants will have to propose an outcome for it to be included in the next round of the process.

At the end of each round, responses will be summarised into two groups: (1) patient participants and (2) all other participants. To be retained into the second round of the e-Delphi process, outcomes will require 50% or more of the respondents in either stakeholder group score it 7 to 9 and fewer than 15% score it as 1 to 3.

Participants who completed round 1 will be invited to complete round 2. Participants will be reminded of their own scores from the previous round for each outcome. They will also be informed of the percentage of individuals from each stakeholder group who rated each of scores 1 through to 9. Participants will be invited to rescore each of the outcomes remaining in the e-Delphi process. They will be advised that they do not need to change their score if they do not want to.

Although we envisage that three rounds may be necessary, we will stop the process following the second round if we reach consensus of 70% or more of the respondents in both groups scoring the remaining outcome’s inclusion as critical (7 to 9) and fewer than 15% score the outcome as not important (1 to 3). If these consensus criteria are not achieved for all outcomes following the second round, the e-Delphi process will be re-run for a third time. Feedback will again be provided to all participants, with individual scores and the percentage of individuals from each stakeholder group that rated each of scores 1 through to 9. Prior to the end of round 3, reminders will be sent to those participants who completed round 2 but have not completed round 2 after 2 weeks.

The remaining outcomes will be taken forward to a face-to-face meeting for discussion with key stakeholders.

#### Face-to-face meeting

At the end of the second round of the e-Delphi process we will ask each participant if they would be willing to take part in a face-to-face meeting to discuss the findings of the process following completion of the e-Delphi. We will reconfirm their willingness if a third round is required. This will be a nominal group meeting held in Dundee, involving 11 participants with representation from each stakeholder groups. Participants will be chosen at random from those expressing willingness to attend. The meeting will be co-facilitated by the lead researcher who is a clinician, as well as an independent methodologist who has experience with group consensus meetings. A detailed protocol will be finalised after the last e-Delphi round.

### Dissemination

To help increase the uptake of the COS we will engage with relevant groups such as the Cochrane Oral Health Group, the British Society of Periodontology, the European Federation of Periodontology, the American Academy of Periodontology, the International Association for Dental Research Periodontal Research Network, guideline development groups including the National Institute for Clinical Excellence, the Scottish Intercollegiate Guideline Network and the Scottish Dental Clinical Effectiveness Programme, journal editors, trial registries and major funding bodies such as the UK National Institute for Health Research (NIHR) Health Technology Assessment (HTA).

## Discussion

Increasingly, there is a greater understanding and emphasis being placed on research being accessible and relevant to all key stakeholders [14]. Historically, the outcome measures used in clinical trials have been chosen largely by the trialists and academics. A number of recent Cochrane systematic reviews have highlighted the need to include patient, clinician and economic-orientated outcomes in future studies [15, 16]. This COS development process and dissemination will start to raise awareness of outcome measure selection and the impact that the selection decisions can have on the synthesis and translation of the evidence base. The COMET initiative is developing COSs in a wide variety of health care areas and similar projects have commenced in other areas of dentistry, namely cariology [17].

## Trial status

The review stage of identifying existing outcomes is complete and the e-Delphi process is currently being planned.

## Abbreviations

COMET: Core Outcome Measures in Effectiveness Trials
COS: Core outcome set
NIHR: National Institute for Health Research
EoSRES: East of Scotland Research Ethics Service
SREC: Schools of Nursing and Health Sciences and Dentistry Research Ethics Committee
